# The putative role of gut microbiota in cancer: Cysteine is a pivotal coin

**DOI:** 10.3389/fgstr.2022.966957

**Published:** 2022-08-05

**Authors:** Jacinta Serpa

**Affiliations:** ^1^ NOVA Medical School|Faculdade de Ciências Médicas, Universidade NOVA de Lisboa, Lisboa, Portugal; ^2^ Instituto Português de Oncologia de Lisboa Francisco Gentil (IPOLFG), Lisboa, Portugal

**Keywords:** gut microbiota, cancer metabolism, cysteine reliance, cysteine bioavailability, aging-related dynamics

## Abstract

Tumor metabolism is mandatory for the proper adaptation of malignant cells to the microenvironment and the acquisition of crucial cellular skills supporting the systemic spread of cancer. Throughout this journey, the contribution of the gut microbiota to the bioavailability of nutrients supporting the bioenergetic and biosynthetic requirements of malignant cells is an issue. This review will focus on the role of cysteine ​​as a coin that mediates the metabolic crosstalk between microbiota and cancer. The key points enclose the way cysteine ​​can be made available by the microbiota, by degradation of more complex compounds or by *de novo* synthesis, in order to contribute to the enrichment of the colonic microenvironment as well to the increase of cysteine systemic bioavailability. In addition, the main metabolic pathways in cancer that rely on cysteine ​​as a source of energy and biomass will be pointed out and how the interspecific relationship with the microbiota and its dynamics related to aging may be relevant points to explore, contributing to a better understanding of cancer biology.

## Introduction

In the human organism, several interspecific relationships are constantly in operation, which are established between the different species that make up the microbiota and the human cells of the various organs where it resides. These interspecific relationships are mainly symbiotic in which both partners benefit. This is the case in health, but in disease, there are still some doubts about the role of the microbiota in the pathophysiology, namely, in the context of cancer, at both the organ and systemic levels. Currently, new clues have been proposed, and several studies have been developed to determine the influence of microbiota in cancer initiation, progression, and therapy, as it is extensively reviewed (1–7).

Metabolic adaptation in cancer is undoubtedly an essential requirement for the establishment, growth, and spread of a malignant neoplasm. Cellular plasticity is crucial for the adaptation of the tumor cell to the microenvironment of the organ where carcinogenesis occurs and to the emergence of stress conditions, such as drug exposure. Recent studies prove that cysteine metabolic circuits are a relevant component of the metabolic network, sustaining biosynthesis and bioenergetics and allowing chemoresistance (as reviewed in 8–10). This review intends to confront some of the most recent findings in the field of cysteine metabolism in cancer and the role of the intestinal microbiota in the dynamic balance of the control of cysteine bioavailability and its putative impact on the progression of oncological disease.

## Gut microbiota composition, interplay, and aging-related evolution

Microbiota is defined as a group of microorganisms that live in a given environment, and it includes bacteria, fungi, protozoa, and viruses, even though viruses are not living organisms. Considering the fungal community, a minor component of gut microbiota compared to bacteria (11), the prevalent genera are *Saccharomyces*, *Candida*, *and Cladosporium* (11–14), with *Candida albicans* the most frequently found in feces of healthy individuals (15). Nevertheless, *C. albicans*, like other intestinal yeasts (16), also presents an opportunistic behavior pattern, being implicated in the development of some infectious diseases (11). Albeit the main studies dedicated to gut microbiota are focused on bacteria, it is known that fungi are important in microbiota reestablishment and equilibrium, immune control, and gut protection (17, 18). The role of fungi and bacteria in the immune response is similar, and these two populations interact and control their own density (16). Importantly, gut fungi seem to be pivotal not only in gut physiology but also in other organs physiology such as the liver, brain, lungs, and kidney (16). Since fungi present specificities that are not deeply explored in cancer, this review will be mainly focused on the bacterial component of gut microbiota.

**Figure 1:**
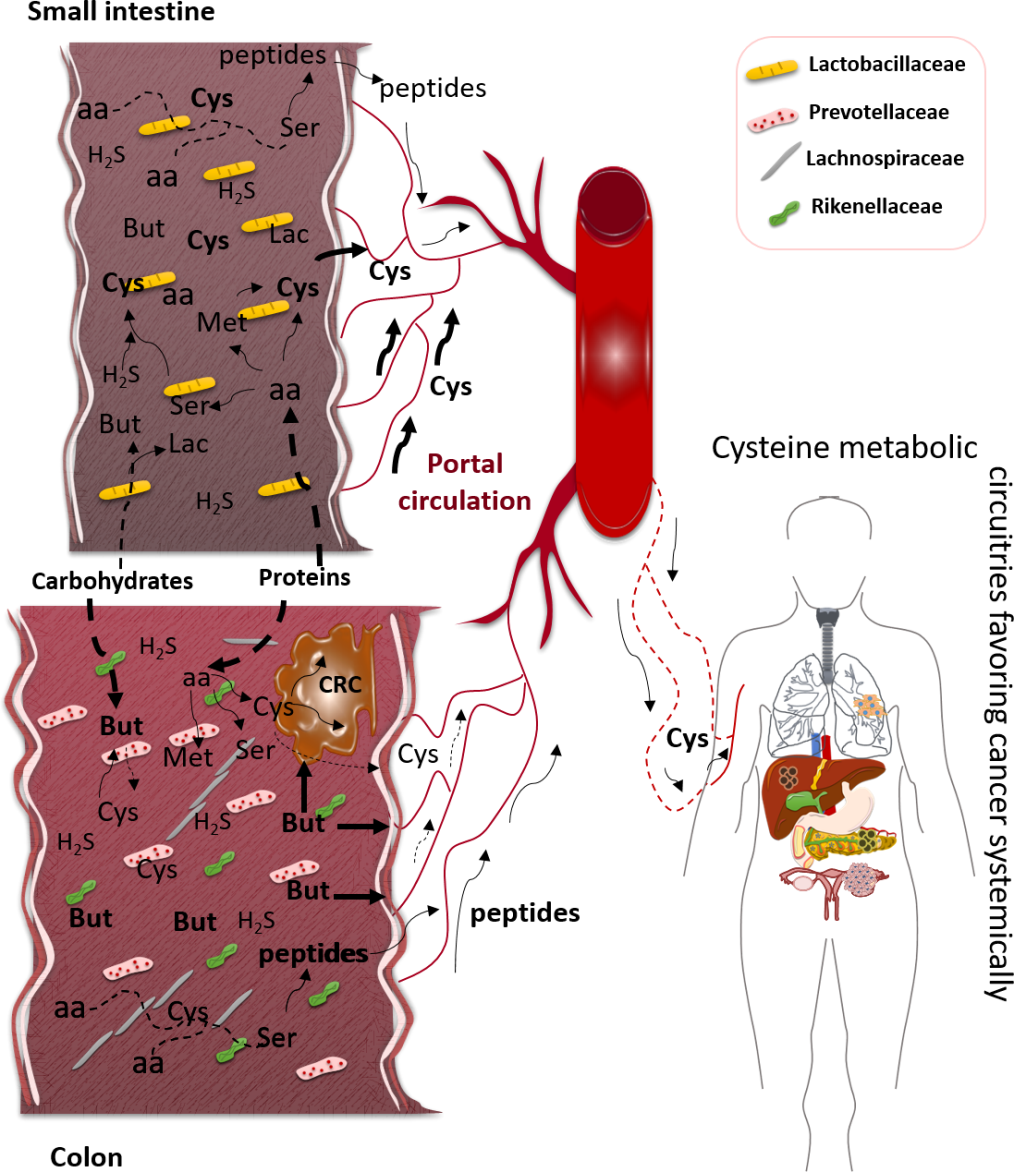
Impact of gastrointestinal (GI) microbiota physiology in cysteine bioavailability, favoring cancer. In small intestine, the prevalent bacterial family is Lactobacillaceae, which can degrade some carbohydrates to generate short-chain fatty acids (SCFAs), namely, butyrate (But), and degrade dietary and host proteins to release amino acids (aa), such as serine (Ser), methionine (Met), and cysteine (Cys). In colon, the dominant bacteria families are Prevotellaceae, Lachnospiraceae, and Rikenellaceae, which represent the most SCFA-producing bacteria, here represented by But. Most parts of dietary proteins are degraded in the colon with the release of peptides and free amino acids (aa). These aa, including Cys, will be mainly absorbed in small intestine since the colon mucosa does not present an efficient absorption of free aa. The peptides can be absorbed by the small intestine and colonic mucosae, and after absorption and distribution through the bloodstream, they can constitute a source of aa, including Cys. Cysteine can result from the degradation of proteins or be synthesized from But or Ser reacting with hydrogen sulfide (H_2_S) or directly from Met. The bulk of H_2_S is Cys-derived, and Cys can also be used to synthesize But. Colorectal cancer (CRC) cells can benefit directly from But, the ones that retain the capacity of metabolizing it and Cys to sustain the metabolic remodeling. Systemically, cancer placed in any organ can benefit from Cys bioavailability, while it enters the blood, to be used as an energy and biomass source, as well as an oxidative stress controller.

The gastrointestinal (GI) microbiota is composed of more than 160 species of bacteria organized in a few phyla, as reviewed by Rinninella etal. (19). Firmicutes and Bacteroidetes phyla represent more than 90% of microbiota, and Actinobacteria, Proteobacteria, Fusobacteria, and Verrucomicrobia phyla account for the major part of the remaining 10%. Firmicutes are mainly composed of *Lactobacillus*, *Bacillus*, *Clostridium*, *Enterococcus*, and *Ruminococcus* genera, and Bacteroidetes are composed of the *Bacteroides* and *Prevotella* genera. GI microbiota is organized from the small intestine to the colon (**Figure 1**), and, based on mouse studies, the small intestine is dominated by Lactobacillaceae, while in the colon, the following prevail: Prevotellaceae, Lachnospiraceae, and Rikenellaceae (20–22).

Different microenvironmental conditions control microbiota representativeness and density, namely, acidity, oxygen availability, the presence of antimicrobial compounds, and the time of transit through the GI tract (20, 22). These variations allow the establishment of facultative anaerobes in the small intestine and of anaerobes, able to digest carbohydrate fibers, in the colon (20, 22).

Along the gut, there are two different microenvironmental niches, the lumen and the mucinous barrier close to the mucosa (mucosal layer), in which the representativity of bacteria is different (23, 24). The formation of a mucous biofilm near mucosa assembles features favoring certain bacteria proliferation and controlling the preferential consumption of particular organic compounds. The impact of these two different niches (mucosal and luminal) on the dynamics of the same bacterial species is clearly described (25). However, microbiota composition works in an individual-specific manner, although a group of designated core bacteria seems to be represented in most individuals. More studies are needed to explore microbiota dynamics in health and disease, and currently, efforts are being made to define the major features required for microbiota to optimize the host systemic metabolism. Most studies trying to map the microbiota distribution and prevalence in the gut are guided by genomics and transcriptomics (26, 27); they revealed that a group of specific bacterial genes seem to be constantly present in the microbiota pool, suggesting that they are crucial for microbiota physiology and they may consequently benefit human physiology. Nevertheless, genomics and transcriptomics are not fully informative to disclose the bacterial physiology and indicate which main pathways support metabolic functioning; thus, biochemical studies are also needed. In addition, research directed to human microbiota is mandatory, since the majority of studies were developed in animal models, and they may not fully represent the human microbiota or the physiology.

The main contributors to microbiota selection and dynamics are energy and biomass sources from diet and host components. Different studies proved the impact of diet on microbiota, mainly relating to dietary patterns in childhood and the typical diet in different spots of the globe with the relevance for a variety of bacterial genera (21, 27, 28). Nonetheless, these studies predominantly consider the enrichment of the intestinal lumen with simple free sugars and carbohydrate fibers, including also the short-chain fatty acids (SCFAs) resulting from the fermentation of the latter ones (27, 29–32). The prevalence of specific energy sources selects bacterial species and contributes to their distribution since they tend to localize in niches enriched with substrates they can degrade. Therefore, species capable of degrading mucins are placed in the mucus layer where they can digest carbohydrates and release the simplest sugars to be used by bacteria without the mucolytic ability (33).

Even the gene expression profile of the host, which determines the composition of the mucus layer, contributes to the selection of bacteria. Intestinal mucus is mainly composed of mucin 2 (MUC2), produced by goblet cells, which is an *O*-glycosylated protein (34). The diversity of *O*-glycans ornamenting MUC2 is conditioned by the genotype and the expression profile of genes encoding glycosyltransferases. Interestingly, the host glycosyltransferase expression profile can be modulated by the action of some species, such as *Ruminococcus gnavus*, *Lactobacillus casei*, and *Bacteroides thetaiotaomicron*, which are somehow able to control the colonization of other bacterial species (35–37). Thus, MUC2 represents an important substrate to which bacteria can adhere and proliferate, but it also harbors important energy and biomass sources for microbiota. The gut biochemical fraction of the microenvironment for sure exerts a crucial selective pressure on microbiota, regulating the balance between bacterial species. Furthermore, the symbiosis established between the microbiota members is also pivotal to control bacteria representativeness and density. Some species produce organic compounds to be shared and used by other species; for instance, *Eubacterium hallii* and *Anaerostipes caccae* can produce butyrate from acetate and lactate, respectively, produced and released by *Ruminococcus bromii* and *Lactobacillus* sp. or *Bifidobacterium* sp. (38–40). Afterward, butyrate is used by human cells as a valuable carbon source and also as a modulator of gut homeostasis and mucosa turnover, due to its role as an epigenetics regulator (41–44).

The impact of aging on evolution and changing of gut microbiota representativeness and diversity is controversial. However, it seems that aging-related alterations in molecular composition and architecture of the intestinal mucosa correlate with a decrease in microbiota diversity (reviewed by (45). Multivariate analysis shows a continuous aging advancement of human GI microbiota along with host aging course (46). Together with this, the metabolic capacity and putative contribution to human physiology will also be remodeled. Hence, the capacity of the microbiota to produce SCFA and degrade starch is reduced with aging, while proteolytic capacity is increased (47, 48). This fact can explain the increased inflammatory process in the intestine of elderly people, due to the lack of the protective effect of SCFA (49), mainly butyrate, whereas the increased capacity to degrade proteins can account for the emergence of cancer beneficial conditions, as it will be discussed later. Furthermore, age-related disequilibrium of the microbiota can favor the installation of novel potentially pathogenic microorganisms.

In gut microbiota equilibrium, cysteine is a major nutrient, not only as a metabolic player but also as a controller of certain pathogenic species, which can overtake microbiota, which is the case of *Clostridium difficile*. *C. difficile* is a nosocomial bacterial responsible for antibiotic-related diarrhea, upon the destruction of the normal gut microbiota (50, 51). In normal conditions, other bacteria control *C. difficile* density by the production of antibiotic compounds and by the regulation of microenvironmental levels of controller nutrients (51–53). Cysteine is one of these nutrients since it functions as a growth and metabolism controller (51–53) and as an inhibitor of the synthesis of *C. difficile* toxin (54). *Escherichia coli* also presents a growth pattern sensitive to cyst(e)ine availability; upon the expression of highly efficient cystine importers, *E. coli* becomes sensitive to oxidative stress because bacteria import excessive cystine, but it is not able to properly metabolize cysteine; thus, this metabolic profile endangers *E. coli* survival (55). This behavior can be triggered by other bacteria as an antibiotic mechanism, and when out of control, it can be a threat to the balance of the microbiota. Nevertheless, *E. coli* strains that are able to metabolize cysteine and produce H_2_S present oxidative stress and antibiotic resistance (56). Hence, cysteine metabolic ability is an important feature to control bacteria density in gut microbiota.

## The main substrates are metabolized by the gut microbiota and contribute to gut enrichment with cysteine and cysteine-related compounds

The focus of this paper is the role of cysteine in microbiota and human cells crosstalk, favoring cancer; therefore, the way cysteine is generated and enriches the gut lumen is an important point to address. Dietary proteins are a source of cysteine, and their digestion occurs along the GI tract, being a considerable proportion (about 10 g) digested in the colon (57). In there, bacteria degrade proteins and use the amino acids for the synthesis of new proteins, peptides, or other organic and inorganic compounds (58). The degradation of the host proteins is also a relevant contribution to cysteine and other amino acid release; for instance, MUC2 presents cysteine-rich domains that are very important for the MUC2-3D structure and the formation of the mucous biofilm (59); thus, MUC2 degradation contributes to cysteine enrichment of gut microenvironment. Moreover, Daniels etal. (60) described that Firmicutes bacteria are able to exclude cysteine from the sequence of cytoplasmic and exported proteins, indicating that this way bacteria are capable of maintaining their resistance to reductant environments since they seem to have acquired an evolutionary skill and they do not rely on disulfide bounds to survive. However, cysteine is used in detoxifying systems, releasing bacteria from damaging compounds, as already described in some Firmicutes genera such as *Staphylococcus* that used bacillithiol (BSH)-related detoxifying systems (61). Therefore, this detoxification process recycles cysteine and may be one more mechanism accounting for cysteine enrichment of luminal gut fraction.

From carbohydrates, the SCFA is the most relevant end product to be absorbed in the human gut and also to be used by other bacteria. The most abundant SCFA are acetate, propionate, and butyrate, and among them, butyrate plays an important role in human physiology, as it is a valuable energy and biomass source, but it is also an epigenetic regulator, controlling gene expression (62). Importantly, butyrate can also be synthesized from amino acid fermentation (**Figure 1**); there is a pivotal metabolic link between butyrate and cysteine since cysteine fermentation is a way of butyrate production (63). In health, butyrate is important in cell renewal, but in cancer, butyrate impacts cell proliferation control and activation of cell death. Thereby, butyrate protects the organism from cancer due to its action as a histone deacetylase inhibitor (HDACi) (64). However, it is described that cancer cells are sensitive to butyrate, as it will function as a HDACi and induce cancer cell death. Nonetheless, some alterations in the microbiota or the metabolic fitness of cancer cells may affect the butyrate-protective outcome. Of note, colorectal cancer patients present a microbiota with diminished representativeness of butyrate-producing bacteria (65); in addition to this, cancer cells that retain the ability to fully metabolize butyrate or are able to adjust their metabolism due to butyrate exposure can escape from cell death control and benefit from butyrate as a carbon and energy (66, 67). Autophagy has also been demonstrated to underlie butyrate resistance in cancer cells (68). Importantly, the synthesis of cysteine from butyrate and hydrogen sulfide (H_2_S) by gut microbiota is not proven, but a study exploring the *in silico* relation of gout arthritis and microbiota physiology indicates that butyrate-producing bacteria are mainly responsible for cysteine production (69). Since other SCFAs, such as acetate, are implicated in cysteine production (69, 70), maybe butyrate can be an undisclosed cysteine source (**Figure 1**).

Additionally, H_2_S, which mainly results from cysteine degradation (71), is a very important player in gut microbiota physiology, and its metabolism can be helpful to understand cysteine microbiota:host interdependency (62) since H_2_S is also a substrate for the synthesis of cysteine by microbiota (72). As reviewed by me (9), in humans, cysteine metabolism is deeply connected with one-carbon metabolism having cobalamin (vitamin B12) as a central compound in the intercross spot between folate and methionine cycles. Importantly, 31% and 37% of daily reference intake of, respectively, cobalamin and folate are estimated to come from gut microbiota (73). In this context, serine and methionine, two important players in one-carbon metabolism, are also connected with cysteine synthesis in gut microbiota. It was described that some strains of *L. casei* are able to synthesize cysteine from serine and H_2_S (72) and methionine (74). As mentioned before, Lactobacillaceae is a prevalent group of bacteria resident in the small intestine where enterocytes are fully capable of absorbing amino acids (**Figure 1**).

## Impact of gut microbiota physiology on cysteine bioavailability

The bioavailability of a nutrient is the pool of this nutrient that is systemically available to be used by the whole-body cells. In the scope of this review, it is important to summarize the contribution of microbiota not only for the enrichment of cysteine in the intestinal microenvironment but also for whether this enrichment can contribute to cysteine bioavailability.

Dietary and host proteins are, upon degradation, important sources of cysteine (**Figure 1**). Hence, cysteine resulting from dietary and host proteins degradation could be conceptually absorbed by colonocytes, contributing to cysteine bioavailability. Nonetheless, it seems that mammalian colon mucosa is not proficient in absorbing amino acids (75), despite epithelial colonic cells expressing a representative panel of amino acid transporters (75). Unfortunately, as reviewed by van der Wielen and co-authors (75), most studies analyzing the expression of amino acid transporters were performed at the transcriptional level, not ensuring the expression of the functional protein and not allowing the evaluation of the cellular localization of these receptors. Nevertheless, if amino acids are efficiently absorbed in the colonic mucosa, meaning if they in fact enter the colonocytes and are directed to the blood, they follow a subcellular route different from that of the small intestine epithelial cells. In the small intestine, amino acid absorption occurs in the apical membrane of the cell, and their release into the bloodstream is performed through the basolateral cell membrane, while studies in pigs and horses showed that in colonocytes, the transport of lysine is only detected in the apical cell membrane without detecting how these amino acids can reach the blood (76). As indicated, in the small intestine, oligopeptides resulting from proteins that were degraded by gastric and pancreatic enzymes (pepsins and proteases) are subsequently digested by peptidases in the brush border of the intestinal wall, and free amino acids are further transported into intestinal cells, follow an intracellular circuit, exported though the basolateral membrane, and canalized into the blood circulation (77, 78). As mentioned above, butyrate can be synthesized from cysteine fermentation (63). Butyrate is mainly produced by Firmicutes species (63, 79), and interestingly, upon aging, these bacteria become less representative in gut microbiota (e.g., *Faecalibacterium prausnitzii*) (48), suggesting that aging by modulating microbiota density and representativeness can decrease the protection of colonic microenvironment against cancer since butyrate concentration decreases together with its anti-cancer effect. Furthermore, the decreased rate of butyrate production can contribute to the accumulation of cysteine in the gut lumen and consequently increase the absorption of cysteine by epithelial cells. Once again, the absorption capacity of colonocytes needs to be explored, since the majority of studies analyzed the absorption of amino acids by indirect methods, measuring preferentially the amount of absorbed nitrogen and not specifically the amino acid-derived nitrogen (80–84). This makes it difficult to determine the contribution of microbiota-released or microbiota-synthesized amino acids for systemic bioavailability, including cysteine. Furthermore, the studies dedicated to the physiological control of amino acids in the gut are antique; it is in fact a requirement to perform new studies with more sensitive and accurate methods.

Bacteria in microbiota also use free amino acids to synthesize peptides (85), which makes part of amino acid turnover pathways, but it also favors alternative ways for colonocytes to take up amino acids without depending on specific amino acid transporters. Cystine is the dipeptide of cysteine, and it seems that most parts of cysteine may be transported across the cell membrane as cystine, mainly mediated by xCT, a glutamate/cystine antiporter. The xCT is expressed in a normal colon and may be a quite specific way of absorbing cysteine from the colonic lumen (86). Likewise, the peptide transporters (PepT) can be an alternative route to compensate for the inefficiency of colonocytes to uptake free amino acids. For instance, PepT1 is one of the most studied and is responsible for the transport of various peptides resulting from diet and putatively from microbiota metabolism (87, 88). In fact, the carrier-mediated absorption of peptides accounts for the major fraction of amino acids absorbed in the gut (89, 90). The inclusion of free amino acids in di- and tri-peptides by microbiota facilitates their import by colonocytes that are unable to transport free amino acids; this is also true for cysteine. Glutathione, a tripeptide of glutamate, cysteine, and glycine, from diet seems to be directly absorbed in the intestine (91), and it is a valued source of cysteine. Additionally, gut microbiota produces glutathione that is absorbed and exerts a great impact on the human body’s antioxidant control (92). Again, as demonstrated in ovarian cancer, glutathione turnover and cysteine metabolic reliance are crucial to sustaining the adaptive capacity of cancer cells as well as chemoresistance (93–95).

Regarding human cysteine bioavailability, it is thought that the bulk of absorbed amino acids come through enterocyte absorption, in the small intestine, and amino acids in the colonic lumen will be mainly used for bacteria metabolism or may be absorbed as di- or tri-peptides but very few as free amino acids.

## Cysteine metabolic circuitries favoring cancer

**Figure 2:**
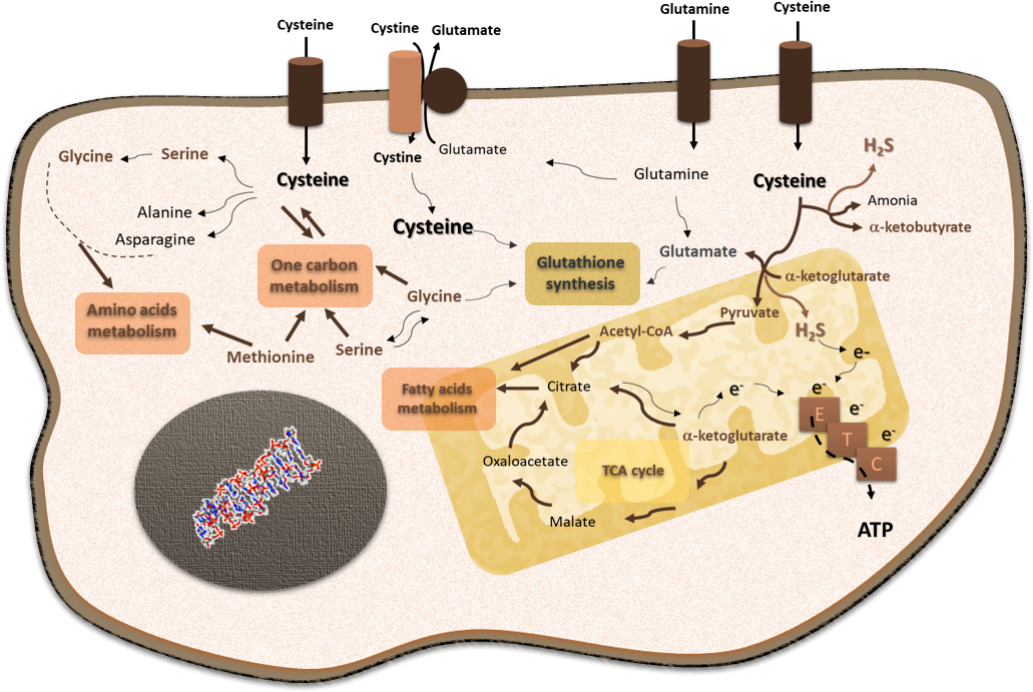
Cysteine is a core player in cellular functioning, supporting its pivotal role in cancer cell metabolism. Cysteine is imported as cystine or as cysteine. Cysteine plays a pivotal role in cancer: it is incorporated in glutathione, a reactive oxygen species (ROS) scavenger; upon degradation in cytosol or mitochondria, cysteine supplies carbon and energy metabolism through fatty acids and amino acid syntheses, tricarboxylic acid (TCA) cycle, one-carbon metabolism, and the production of ATP through the electron transport chain (ETC), and it contributes to sulfur and energy production as a generator of hydrogen sulfide (H_2_S) and a donor of electrons (e−) to the ETC.

Cysteine occupies a core position in cancer cell metabolism (**Figure 2**). As described, cysteine is an important player in oxidative stress control, as a free amino acid or included in the glutathione molecule. The control of the redox cellular state is a key ability allowing the maintenance of the metabolic flow (96–99). On the one hand, the cysteine metabolic reliance provides increased glutathione levels and an efficient turnover, which permits cancer cells to cope with stressful conditions, such as hypoxia and drugs (93, 94). Hence, cysteine fitness constitutes a relevant mechanism of chemoresistance for cancer cells accounting for their capacity of escaping from the action of oxidative and alkylating anti-cancer drugs (93, 94, 100–106). On the other hand, cysteine is posited as valuable bioenergetics and biosynthetic source, able to replace core metabolic elements, such as glucose and glutamine. The cysteine metabolic network depends on its versatility as sulfur and as a carbon source. This network presents three main steps: 1) cysteine transport across the cell membrane, 2) cysteine catabolism, and 2) cysteine anabolism.

### Cysteine import

Cysteine uptake is mediated by specific transporters, and cysteine can enter the cell as a free amino acid or as a dimer, cystine (107–111). The increased expression of xCT is described in cancer as being associated with more aggressive and chemoresistant phenotypes (100, 107, 112–116), and despite that most of these studies concern glutamate export, the role of cysteine uptake in the maintenance of those tumors can be assumed since for glutamate to leave the cell, cyst(e)ine entrance is mandatory. Although cystine is the main form taken up by cancer cells, cancer cells can also import cysteine directly (117) by overexpressing specific cysteine transporters, namely, the amino acid transporter 3 (EAAT3; SLC1A1 gene) (Nikolaos Pissimissis, Efstathia Papageorgiou, Peter Lembessis, Athanasios Armakolas, 2009; 108, 118) and the alanine-serine-cysteine-transporter 2 (ASCT2; SLC1A5 gene) (119–121). Since these transporters also mediate the transfer of other amino acids, their expression in the cancer context is not always associated with cysteine dependence. Furthermore, considering ferroptosis, a newly described cell death process, the intracellular levels of cysteine are crucial for the maintenance of glutathione to ensure the lipid peroxide scavenging. This process is catalyzed by glutathione peroxidase 4 (GPX4), which uses glutathione as a substrate. This way, xCT is associated with resistance to ferroptosis (122).

### Cysteine catabolism

Cysteine degradation depends on four enzymes: cystathionine β-synthase (CBS), cystathionine γ-lyase (CSE), and 3-mercapto-pyruvate sulfurtransferase (MpST), which acts after cysteine aminotransferase (CAT) (123). Cysteine catabolism generates H_2_S and different organic compounds, such as pyruvate, serine, and α-ketoglutarate (124–129). H_2_S functions as an electron donor to the electron transport chain (ETC) (124, 130, 131), and it also acts as a signaling molecule, regulating cellular processes relevant to cancer, namely, cell survival, proliferation, and angiogenesis (93, 94, 132, 133). The organic compounds generated from cysteine degradation can be canalized into different metabolic pathways, such as the tricarboxylic acid (TCA) cycle, one-carbon metabolism, amino acids, and fatty acid syntheses.

Cysteine can also be a source for pathways that are typically related to glucose, such as gluconeogenesis and the pentose phosphate pathway (PPP). Cysteine may be used to synthesize glucose through gluconeogenesis, as it originates from pyruvate and the gluconeogenic amino acid, alanine. This way, cysteine contributes to the transient pool of glucose within the cell. A very recent study showed that in fact cysteine is used to generate alanine and lactate (95), mainly synthesized from pyruvate that presents a transient permanence in the cell. Gluconeogenesis is currently receiving some attention in cancer, as a way of increasing glucose yield in the cell without depending on glucose bioavailability and transport (reviewed by 9, 10). Another glucose-dependent pathway is PPP, which can benefit from the link between glucose and cysteine metabolism by using cysteine-derived glucose. Moreover, the inhibition of the final step of gluconeogenesis prompts glucose-6-phosphate into PPP. Furthermore, cysteine contributes to glucose metabolic flow by controlling the redox state of the cell, since the pivotal enzymes of gluconeogenesis and PPP, respectively, PCK1 (phosphoenolpyruvate carboxykinase 1) and G6PD (glucose-6P-dehydrogenase), are directly regulated by Nrf2, a master regulator of redox control, which is sensitive to oxidative stress that is consequently dependent on cysteine circuitries (134, 135). In addition, PPP is also a player in redox control having cysteine-derived glutathione as an intermediate (136). Therefore, cysteine is a valuable carbon source used by cancer cells to support their energy and biomass demands.

### Cysteine anabolism

Cysteine synthesis occurs through the transsulfuration pathway (TSP), and it depends on the sequential action of CBS and CSE, which are also involved in cysteine catabolism, as mentioned. The TSP is a metabolic branch that sprouted from the deviation of homo-cysteine from the methionine cycle in one-carbon metabolism (137). Homo-cysteine is condensed with serine by CBS, and the resulting cystathionine is hydrolyzed by CSE, giving rise to cysteine, ammonia, and α-ketoglutarate (138). Here, a link between cysteine metabolism and the TCA cycle can be found through α-ketoglutarate. The degradation of oxidized glutathione (GSSG), through the γ-glutamyl cycle, will allow the recycling of its three components: glutamate, cysteine, and glycine. GSSG exits the cell, and its degradation is catalyzed by γ-glutamyl transpeptidase (GGT) located at the external face of the cell membrane (139). After glutamate is released, the cysteinylglycine dipeptide can re-enter the cell through PEPT2 and be converted to cysteine and glycine upon the action of dipeptidases (140), or it can be degraded by aminopeptidase N (APN), and cysteine and glycine are again available to re-enter the cell (141). Cysteine synthesis is linked to different amino acid metabolism; for instance, glycine and serine are glutamine-derived and are important suppliers of the folate cycle from one-carbon metabolism (as reviewed by 9).

The enzymes involved in both cysteine catabolism and anabolism, CBS and CSE, are frequently associated with malignancy and more aggressive cancer phenotypes (124, 127, 142–147), suggesting that at least one of the two pathways are relevant in cancer, and they might be working simultaneously as a way to keep on moving the metabolic cellular network. Concerning MpST, little is known about its association with cancer; however, there are some indications provided by *in vitro* assays with pharmacological inhibitors and silencing approaches, suggesting that this enzyme can be critical for cancer cell proliferation, bioenergetics, and cell signaling (125).

The metabolism of cysteine associated with its transport is composed of an endless circle moving a huge number of intermediaries that can be made available for the most varied metabolic pathways (**Figure 2**). In this way, cysteine provides the malignant cell with plasticity and adaptive capacity, which will benefit the progression of the disease. The enrichment of the tumor microenvironment and biological fluids in cysteine is a strong indication that this is true. Thus, the systemic bioavailability of cysteine is strictly necessary for the success of the oncological disease, and all contributions to increase these indices will contribute to the poor prognosis of the disease.

## Gut microbiota affects cancer progression by controlling cysteine bioavailability, also upon aging

In the human gut, bacteria work together, and the metabolic symbioses are important components of gut biological dynamics. The metabolic expertise of different bacterial species and strains (79) keeps on the metabolic flow based on organic compounds sharing, in which some compounds are produced by certain bacteria to be used by other bacteria, contributing to the maintenance of a healthy variability and density of microbiota, ideally preserving corresponding metabolic profiles. The metabolic dynamics of gut microbiota influence human health and disease.

Metabolomics is used to assess the metabolic interplay between microbiota and host by metabolically mapping different human body fluids. Most studies on the gut microbiota metabolome are designed to investigate dysbiosis, which means disease-related metabolic profiles (148). Actually, the gut microbiota metabolome helps to define metabolic profiles that may be useful to distinguish between unhealthy and healthy individuals (reviewed by 149). Different studies have found metabolic signatures associated with inflammatory, metabolic, and neurological/neurodegenerative disorders and cancer (150–154). The studies dedicated to cancer presented promising results associating gut microbiota and metabolome with disease specificities. In colorectal cancer, associations were found with microbiome and metabolome in different disease stages (155). Genomics and metabolomics data reported that the gut microbiota regulates the immune response in hepatocellular carcinoma (156). Trials were proposed to explore the diagnostic and prognostic values of the definition of gut microbiota metabolome in breast cancer (157). Recently, Hermida etal. (158) presented a predictive study of therapy response using The Cancer Genome Atlas (TCGA) datasets from different cancer types; the authors concluded that it is possible to predict in naive biopsies, which will be the therapy outcome of tumors based on tumor microbiome RNA-seq and whole-genome sequencing analyses.

The microbiota and cancer interplay is an important connection to explore, since it encloses a possible contribution of microbiota functional network to cancer metabolic reliance, favoring systemic disease progression. We and others described that cancer patients’ body fluids are enriched in cysteine, which can come from endogenous synthesis, transsulfuration pathway, and protein degradation or by increased intestinal absorption of cysteine intestinal content that originated from diet and microbiota metabolism.

Cysteine is a very important compound in cancer metabolism from different perspectives, and studies have demonstrated that cysteine is the main thiol in the biological fluids of cancer patients. In ovarian and pancreatic cancers, cysteine was shown to be a relevant carbon source, sustaining bioenergetics and biosynthesis, as well as a pivotal H_2_S source needed for ATP production (93, 94, 159). Furthermore, and considering all the cancer progression journey, chemoresistant cancer cells exhibit cysteine metabolic reliance accounting for increased glutathione levels and consequently augmenting the scavenging capacity of reactive oxygen species (ROS) needed to cope with oxidative stress, which simultaneously will abrogate the cytotoxic action of most drugs conventionally used to treat cancer (8). The tumor and the systemic microenvironment are rather important in carcinogenesis and disease progression, and assuming the relevance of microbiota, we must define different scenarios since gut-located tumors will directly access organic compounds generated by microbiota, whereas tumors developed in other organs need those organic compounds to reach the bloodstream. Different contributions are needed to increase cysteine intestinal absorption mediated by membrane transporters. Since they have a regulated expression, the substrate availability, in this case cysteine-enriched gut microenvironment, is a stimulus for the expression of transporters by epithelial and cancer cells. As mentioned, little is known about the expression dynamics of cysteine transporters at the protein functional levels. This would be a prevailing step in setting up the contribution of microbiota for cysteine bioavailability in health and disease.

Considering colorectal cancer, cancer cells placed in a cysteine-rich microenvironment might benefit from cysteine without being dependent on the absorptive capacity of colonic mucosa (**Figure 1**). Cancer cells express different cyst(e)ine transporters that mediate its uptake directly from the colonic lumen. In colorectal cancer cells, a pivotal cyst(e)ine transporter is xCT and a mechanistic loop sustaining xCT expression involve cysteine-derived H_2_S-dependent persulfidation of OTUB1, the deubiquitinase that regulates xCT stabilization, suggesting cyst(e)ine through the metabolic circuitries control the expression of its own transporter (160). Moreover, xCT is also expressed in the normal colon, but it was demonstrated that its overexpression in colorectal tumors is associated with the activation of MELK oncogene and Pi3K and RAS pathways, being xCT pharmacological blockade a way of affecting cancer cells tumorigenesis (5, 111). Actually, xCT was proposed as a biomarker for colorectal cancer recurrence (161), reinforcing the role of xCT and the need for cysteine as an important metabolic hallmark in cancer. However, other types of cancer developed in different organs present specificities that encompass the need for cysteine absorption. For sure, the pool of cysteine absorbed in the small intestine (162, 163) will benefit cancer cells with metabolic reliance on cysteine, being a cysteine pool that originated from diet and microbiota metabolism (**Figure 1**). Outside of the gut, the cysteine pool in the tumor microenvironment comes from the bloodstream, and the metabolic activity of cancer and non-cancer cells shares the same niche. The cysteine reliance of cancer cells implies a frequent uptake of cyst(e)ine, even when the endogenous cysteine synthesis is occurring, thereby expressing different cyst(e)ine transporters, a cancer cell can manage the import of cysteine according to its own metabolic state and needs (9).

The impact of cysteine on cancer advance is also seen in cancer patients’ survival and cachexia. Cachexia is a life-threatening condition associated with different diseases and causes extreme weight loss and muscle wasting (164). Cachexia is a marker for poor cancer prognosis, occurring in about 80% of patients and accounting for at least 20% of cancer-related deaths (164–166). A study dedicated to the cachexia effects in a GI cancer cohort revealed that patients who received cysteine supplementation in parenteral nutrition had shorter overall survival as compared to those who did not receive cysteine (167). In the same study, the authors demonstrated that cyst(e)ine deprivation suppresses the growth of colorectal xenograft tumors and potentiates the oxaliplatin effect, and the mice did not lose weight (167).

In brief, cysteine interdependence of microbiota and cancer cells can be seen at least in two ways: 1) cysteine made available by microbiota can be used by cancer cells as a metabolic source, and 2) cysteine-derived compounds, such as glutathione and H_2_S produced by microbiota, can be used by cancer cells as antioxidants and as important players in metabolic flow and energy production. As depicted in the review by Bonifácio etal. (10), cysteine follows different circuitries in the metabolic network, serving as a metabolic coin but also as a regulator of metabolism, accounting for cellular and body homeostasis. Cysteine versatility in cancer received recently more attention since new studies disclosed the panoply of pathways that are dependent on cysteine bioavailability, emphasizing that the cysteine metabolic map is a pivotal component of cancer cells’ metabolic remodeling in order to cope with stressful conditions imposed by the tumor microenvironment and by severe disturbance of body equilibrium in advanced diseases.

## Conclusions

In this review, several aspects of the intestinal microbiota and cancer duality were addressed, which together demonstrate that there is an opportunity for intervention. The impact of the GI microbiota is decisive in the bioavailability of cysteine in the human body, and this evolves with aging. Once cancer is a group of diseases mostly potentiated by aging, it is natural that cysteine and its various valences play a leading role in cancer promotion and progression.

This compilation also serves to reflect on the latest dietary practices, highly enriched in protein and low in carbohydrates. The prevailing idea that glucose is the cancer nutrient misleads the total elimination of carbohydrates in some diets recommended for cancer patients since amino acids are the main substitutes for glucose. Glutamine has long been known to be the main glucose substitute for sustaining cellular respiration, with glucose and glycolysis primarily serving biosynthesis. Currently, cysteine has also assumed a leading role in bioenergetics and biosynthesis in the metabolism of malignant cells. In addition, the reduction of carbohydrates also significantly reduces the bioavailability of butyrate and its anti-cancer protection factor.

More studies are needed to reinforce the role of the gut microbiota in the metabolic drift that accompanies aging, in which cysteine is one of the most important coins. Thus, it will be possible to establish protocols to monitor and adjust the microbiota to the aging process. A pharmacological alternative that could be tested is blocking cysteine absorption (transport of cysteine in the intestinal mucosa); considering cysteine is not an essential amino acid, the impact in normal cells would be reduced. However, this inhibition must be performed with formulations that act only at the intestinal level without being absorbed, as this could have a deleterious impact on the metabolic dynamics of the body. Therefore, different strategies can be followed in an attempt to avoid the establishment of conditions that may be more favorable to the progression of cancer.

## Author contributions

The author confirms being the sole contributor of this work and has approved it for publication.

## Funding

The institutions are funded by *Fundação para a Ciência e Tecnologia/Ministério da Ciência*, *Tecnologia e Ensino Superior* (FCT/MCTES, Portugal) through national funds to iNOVA4Health (UIDB/04462/2020 and UIDP/04462/2020) and the Associated Laboratory LS4FUTURE (LA/P/0087/2020).

## Conflict of interest

The author declares that the research was conducted in the absence of any commercial or financial relationships that could be construed as a potential conflict of interest.

## Publisher’s note

All claims expressed in this article are solely those of the authors and do not necessarily represent those of their affiliated organizations, or those of the publisher, the editors and the reviewers. Any product that may be evaluated in this article, or claim that may be made by its manufacturer, is not guaranteed or endorsed by the publisher.
